# Down-Regulation of Negative Emotional Processing by Transcranial Direct Current Stimulation: Effects of Personality Characteristics

**DOI:** 10.1371/journal.pone.0022812

**Published:** 2011-07-29

**Authors:** Cleofé Peña-Gómez, Dídac Vidal-Piñeiro, Immaculada C. Clemente, Álvaro Pascual-Leone, David Bartrés-Faz

**Affiliations:** 1 Departament de Psiquiatria i Psicobiologia Clínica, Facultat de Medicina Universitat de Barcelona, Barcelona, Spain; 2 Departament de Psiquiatria i Psicobiologia Clínica, Facultat de Psicologia, Universitat de Barcelona, Barcelona, Spain; 3 Berenson-Allen Center for Non-Invasive Brain Stimulation, Beth Israel Deaconess Medical Center, Harvard Medical School, Boston, Massachusetts, United States of America; 4 Institut Universitari de Neurorehabilitació Guttmann-UAB, Badalona, Spain; 5 Institut d'nvestigacions Biomèdiques August Pi i Sunyer, Barcelona, Spain; University of Groningen, The Netherlands

## Abstract

Evidence from neuroimaging and electrophysiological studies indicates that the left dorsolateral prefrontal cortex (DLPFC) is a core region in emotional processing, particularly during down-regulation of negative emotional conditions. However, emotional regulation is a process subject to major inter-individual differences, some of which may be explained by personality traits. In the present study we used transcranial direct current stimulation (tDCS) over the left DLPFC to investigate whether transiently increasing the activity of this region resulted in changes in the ratings of positive, neutral and negative emotional pictures. Results revealed that anodal, but not cathodal, tDCS reduced the perceived degree of emotional valence for negative stimuli, possibly due to an enhancement of cognitive control of emotional expression. We also aimed to determine whether personality traits (extraversion and neuroticism) might condition the impact of tDCS. We found that individuals with higher scores on the introversion personality dimension were more permeable than extraverts to the modulatory effects of the stimulation. The present study underlines the role of the left DLPFC in emotional regulation, and stresses the importance of considering individual personality characteristics as a relevant variable, although replication is needed given the limited sample size of our study.

## Introduction

The control process of manipulating when, where, how, and which emotion is experienced or expressed has been termed emotion regulation, and can occur automatically or consciously [1]. Emotions play a key role in social behavior as well as in modeling different cognitive functions such as memory [2], decision making [3], and attention [4]. How emotions are regulated determines the proper behavioral responses to achieve different goals at intra- and interpersonal levels [5], [6]. The importance of emotion regulation is highlighted by the fact that inappropriate emotional responses may lead to a disruption in behavior, and in extreme cases to severe psychopathology [7], [8].

Through connections with subcortical nuclei such as the amygdala, the nucleus accumbens, the ventral striatum and the dorsal raphe nucleus, the medial, ventral and lateral portions of the prefrontal cortex (PFC) play a key role in both negative and positive emotional regulation [9], [10], [11]. Specifically, the dorsolateral PFC (DLPFC) is one of the brain regions implicated in emotional processing, particularly during down-regulation of negative emotional conditions [12]. Increased activity in the DLPFC was reported in fMRI studies during awareness of neutral stimuli and suppression of fearful stimuli (faces) [13], and during the processing of positive emotional stimuli in comparison with the evaluation of neutral and negative ones [14]. Furthermore, a number of studies using electrophysiological and/or functional neuroimaging techniques have reported consistently increased activity in the DLPFC under reappraisal conditions (modifying the intensity of emotional stimuli using cognitive strategies; [15], [16]).

Emotional regulation is a phenomenon subject to major inter-individual differences [7]. However, little is known about the mechanisms that might underlie this variability. Some findings point to baseline neurofunctional or psychophysiological characteristics, such as high levels of baseline left prefrontal activation [17] or increased salivary cortisol levels [18], as important variables linked to the individual capacity to regulate emotions. Emotional responsiveness is also known to be influenced by psychological aspects such as personality traits [19], [20]. For example, individuals who record high scores on extraversion measures, tend to be optimistic and enjoy social contact, report higher positive emotions in their daily life and are more likely to express their emotions, both positive and negative. On the other hand, individuals who score high on neuroticism and tend to be anxious, worried, sad, tense and apprehensive, report higher negative emotional experiences and more negative emotions than less neurotic individuals [1], [21].

Personality traits have been shown to be relatively consistent and stable over time, and evidence linking biological mechanisms to various dimensions of personality is beginning to accumulate. Personality has a moderate degree of heritability, and particular gene variations influence the expression of personality characteristics [22]. Regardless of the relevance of genetic factors in combination with environmental variables in determining the configuration of personality differences, the expression of personality (like any complex psychological construct) also appears to be amenable to study on the basis of neural network dynamics. Brain imaging studies have revealed different patterns of brain activity in response to emotional stimuli as a function of individual differences in anxiety, harm avoidance, extraversion, phobic fears, or attachment style [23], [24], [25]. Specifically, extraversion has been associated with amygdala activity in response to happy faces [23], reduced prefrontal resting-state activity [26] or lateral prefrontal BOLD response to cognitive demands [27], while neuroticism has been linked to specific changes in amygdala to dorsomedial PFC and amygdala to anterior cingulate connectivity when processing fearful faces [28], and positively correlated with responses in the anterior cingulate cortex in response to anticipatory fear [29].

Transcranial direct current stimulation (tDCS) delivers continuous weak electrical current through electrodes positioned over the subject's scalp surface. This technique exerts a neuromodulatory effect, shifting subthreshold neuronal membrane potentials in a polarity-dependent manner, increasing (anodal-tDCS) or decreasing (cathodal-tDCS) cortical excitability [30], [31]. The physiological effects of tDCS have been reported to last for about one hour after several minutes of continuous stimulation in humans [30] and have been linked with neurophysiological mechanisms of long-term potentiation and depression [32]. Furthermore, the effects of tDCS can be considered site-specific but not site-limited [33], as functional neuroimaging techniques have revealed changes in metabolic rate [34], [35] or functional connectivity [36], [37] not only under the site of stimulation but also in distant areas presumably connected. Behaviorally, a frequent observation of anodal tDCS over the DLPFC is a transient amelioration in a range of cognitive functions including working [38] and declarative [39] memory, probabilistic classification learning [40], language learning [41] and visual recognition memory [42]. However, despite these clear modulatory effects on both cognition and brain activity, only one previous report [43] has investigated the potential impact of tDCS on emotional processing. In that study, healthy participants viewed seven pictures restricted to human pain situations while receiving 5min of tDCS (2mA) over left-DLPFC, resulting in a decrease in emotional discomfort ratings.

Based on the above-mentioned findings regarding the neural circuitry of emotion regulation, the relevance of individual differences linked to personality characteristics, and the proven capacity of electrical stimulation to modulate cognitive functions and behavior in humans, our study had two main objectives. The first was to investigate whether the application of tDCS over the left-DLPFC modulates emotional valence ratings while subjects evaluate a large number of negative, positive and neutral emotional pictures. The second was to explore whether individual differences in personality traits could modify the influence of electrical brain stimulation on emotional ratings. To do so, we focused on extraversion and neuroticism dimensions. In this regard, there is evidence that high scores in extraversion are associated with a difficulty to suppress expressions of emotion [6]. Further, it was formerly demonstrated from EEG and fMRI studies that high levels of baseline left prefrontal activation are associated with increased capacity to voluntarily suppress negative emotions [15], [17], [44]. In fact, increased baseline levels of activity (left prefrontal and amygdala) during the processing of negative, relative to positive emotional pictures, appear to be characteristic of individuals exhibiting high ratings in introversion [45]. Hence, since behavioral emotional expression seems to be more malleable in introverts than in extraverts and this might be mediated by greater baseline levels of left prefrontal activity, we hypothesized that potentiating excitatory neuromodulatory changes by applying anodal tDCS in this brain region [31], would result in more prominent and noticeable effects during affective picture processing in participants with high scores in introversion than in extraversion. Finally, the up-regulation of negative emotions in subjects with high scores on neuroticism compared to more stable individuals [46] led us to hypothesize that left DLPFC stimulation would mainly modulate negative emotional ratings in these individuals.

## Methods

### Subjects

Sixteen right-handed healthy women (mean age = 22.93, S.D = 4.18) were included in the study. As in previous reports [14], only women were chosen because they are more likely to show strong physiological responses to emotional stimuli than men [47]. Individuals with medical history of psychiatric or neurological conditions, including substance abuse, substance dependence or depression were excluded, using a cut-off score of 13 on the Beck depression inventory [48]. All subjects gave written informed consent to participate in the study, which had been approved by the Bioethics Committee at the Hospital Clínic of Barcelona, Spain.

### Transcranial Direct Current Stimulation

Direct electrical current was applied to the subject's scalp through saline-soaked sponge electrodes (5×7 cm) connected to a battery-driven constant current stimulator, Phoresor PM850 (IOMED, Salt Lake City, Utah, USA). The electrodes were positioned in accordance with the 10–20 international system for electroencephalogram electrode placement. In the main experiment, the anode was positioned centered on F3 (left prefrontal) and the cathode was positioned over the C4 (right motor cortex).

**Figure 1:**
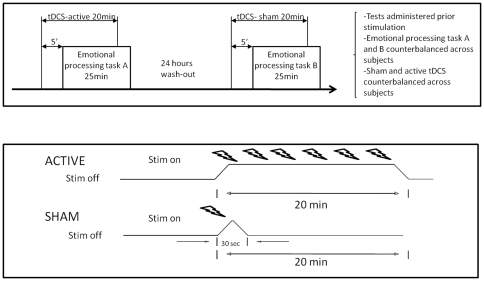
Experimental design. Each subject underwent sham and active stimulation on two different days in a counterbalanced design (see panel A). Two equivalent emotional processing tasks were used (A and B in the figure), which were randomized within subjects. For real tDCS, 20 min of 1mA was continuously applied whereas for the sham stimulation the current stimulator was turned only during the first 30 seconds, to mimic the somatic sensations without actually affecting the underlying cerebral cortex (see panel B).

Active tDCS consisted of a constant current of 1mA applied for 20 min. For sham tDCS, electrodes were positioned as described above, but the current was delivered only for 30 sec and then ramped off (see panel B, figure 1). This method is commonly used by other recent investigations [39], which have shown that in general subjects are unable to distinguish between active and sham stimulation [49].

### Experimental Design

We conducted a randomized, sham-controlled, crossover trial which took place over two separate days. On the first day all subjects performed the emotional valence rating task (see description below). Half of them were randomly assigned to receive sham tDCS, and the other half underwent active tDCS. To control for putative differential practice effects between active and sham conditions, the eight subjects beginning with sham tDCS performed the task under active stimulation on the second day and vice-versa (figure 1).

### Stimuli and procedure

**Figure 2:**
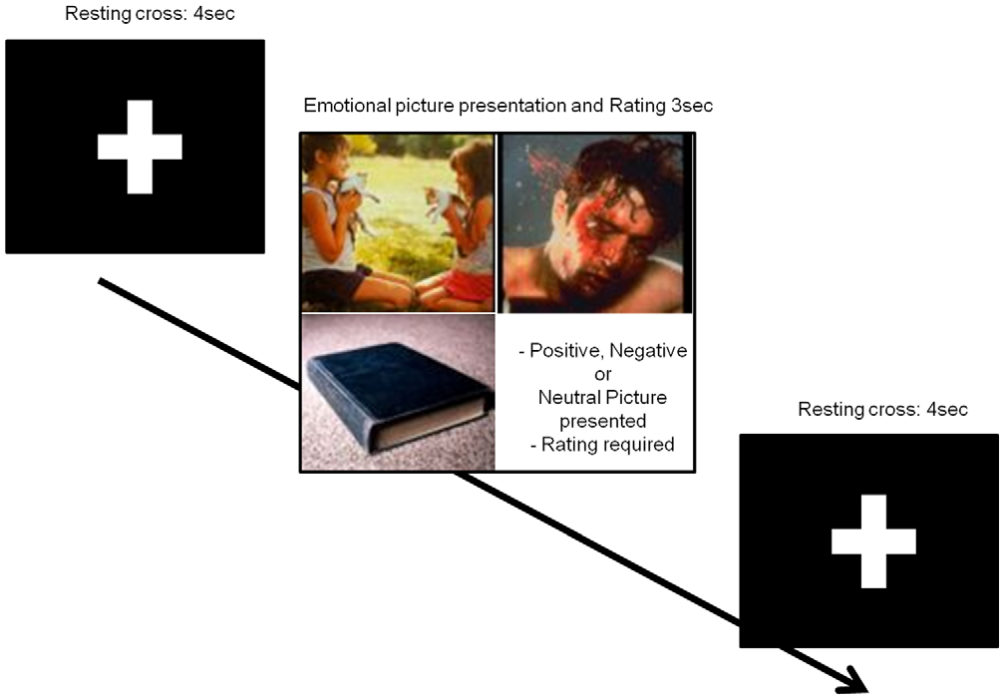
Stimulus presentation protocol and examples of pictures used in the study. Before the presentation of each picture a white cross was shown on the screen for 4 seconds, followed by a positive, negative or neutral picture for 3 seconds, requiring emotional rating by the subject. Then another cross was presented for 4 seconds. Note that although the example contains pictures of the three emotional valences (positive, neutral, negative), during the experiment only one picture corresponding to a single emotional valence was presented at each time.

Stimuli consisted of 180 pictures (60 positive, 60 negative and 60 neutral) selected from the International Affective Picture System (IAPS) picture database [50]. Images were presented using the Presentation software program (version 0.50, Neurobehavioral Systems, 2002; http://www.neurobs.com/) implemented on a laptop computer. The 15 inch screen was held at eye level and at an approximate distance of 50 cm from the subject. Each picture was presented filling the entire screen for 3 sec with an inter-stimulus fixation cross interval of 4 sec (figure 2). The IAPS provides normative values for each stimulus (the ones for young females were considered in the present report) based on the ratings of arousal and valence dimensions. In this context, *arousal* refers to a dimension that varies from calm to excitement, and *valence* refers to a dimension that varies from unpleasant (negative) to pleasant (positive) with neutral in the middle.

In each condition, while under real or sham tDCS, subjects were instructed to evaluate each picture using a 9-point Likert-type scale by pressing a button on a keyboard (1 = negative, 5 = neutral, 9 = positive). Subjects were told to evaluate the pictures according to their subjective emotional perception. The images were presented in such a way that no more than two pictures of the same emotional valence could be presented consecutively. Two equivalent tasks were used in our counterbalanced design regarding condition (sham vs active stimulation) and moment of stimulation (day 1 or day 2, see table 1). The two tasks contained the same number of pictures and were comparable in terms of arousal and valence (see table 1).

**Table 1:** Comparison of tasks A and B in terms of the representation of valence and arousal dimensions.

VALENCE (1-9)/AROUSAL (1-9)			
	TASK A	TASK B	T (sig)
**POSITIVE**	7.34(0.59)/5.55(0.86)	7.28(0.58)/5.32(0.79)	0.63(0.53)/1.35(0.18)
**NEGATIVE**	2.26(0.50)/5.90(0.78)	2.32(0.48)/5.82(0.56)	0.61(0.54)/1.41(0.16)
**NEUTRAL**	5.19(0.45)/3.31(0.64)	5.04(0.38)/3.26(0.52)	1.87(0.07)/0.53(0.60)

Each task consists of 180 stimuli, as described in the main text. Values are given in mean (SD).

Finally, a parallel study using the same experimental design as in the main experiment was undertaken in 9 independent healthy young women (age: 25.80 (5,20)). This second experiment served as a control to verify that anodal, but not cathodal, stimulation exerts an effect on emotional regulation. The polarity of the electrodes was reversed, thus placing the anodal electrode over C4 (right motor cortex) and the cathodal electrode over the F3 (left prefrontal).

### Assessment of other behavioral measures

To investigate putative changes in emotional ratings under active compared to sham stimulation we considered comparisons between each of the valence conditions (positive, neutral, negative) under each stimulation condition as well as changes from sham to active ratings within each valence. Further, in order to control for possible mood effects of tDCS, before and after each session of sham or active stimulation, a series of visual analogical scales (VAS) was administered. The VAS consisted of a 10-cm solid horizontal line (left-edge =  minimal value, right-edge = maximal value) which subjects were required to intersect by drawing a vertical mark reflecting their subjective perception in each of the following states: nervousness, contentment, sadness, hope and annoyance.

We also analyzed potential changes induced by electrical stimulation in the Positive and Negative Affect Schedule (PANAS) [51], a commonly used 20-item self-report questionnaire developed to measure positive and negative affect, as well as in the ‘state’ part of the STAI [52], where subjects are requested to respond to 20 items each with four options of response, reporting personal anxiety levels at the moment of evaluation. Finally, personality was included in the assessment as a potential modifier of the tDCS effects on emotional ratings, as hypothesized above. To do so, we used the NEO-FFI questionnaire [53], a reduced version of the NEO Personality Inventory (NEO PI-R) [54], which includes 60 items (12 items per domain) measuring personality traits such as Neuroticism (N), Extraversion (E), Openness to Experience (O), Agreeableness (A) and Conscientiousness (C).

### Data analyses

The Statistical Package for Social Sciences (SPSS, version 16.0) was used for all the statistical analyses, with a two-tailed p-value <0.05 considered significant. Prior to analyses, all variables underwent Kolmogorov-Smirnov testing. As the null hypothesis could not be rejected in any of the cases, the distributions were assumed to be normal. For the main analysis of this study we performed a three-way repeated-measures (2x3x2) analysis of variance (ANOVA) to test the main effects and the interactions of the following factors over emotional picture ratings: effect of order of stimulation (first day/second day), valence (negative, neutral and positive), and stimulation condition (sham vs. real). Repeated measures ANOVAs searching for main effects and interactions were performed to test any possible effect of stimulation condition (real vs. sham) or administration time condition (before tDCS vs. after tDCS) on VAS, STAI and PANAS variables. Analyses were repeated for the control (cathodal) experiment, and post-hoc paired t-test comparisons (i.e. sham vs. real stimulation within each level of the valence factor) were subsequently performed to investigate the direction of the differences, when significant findings emerged in the ANOVAs. The Huynh-Feldt correction was applied when sphericity assumptions were violated. Finally, a correlation analysis was performed between the magnitude of changes observed in valence rating under real tDCS and personality traits (extraversion and neuroticism), to determine whether individual variability in this latter variable modulates the effects of brain stimulation.

## Results

All subjects completed the entire experiment. None of the subjects evaluated using the NEO-FFI questionnaire in our study obtained values suggesting clinical alterations on any scale. No adverse effects such as pain, skin burns or irritation, or headache were reported during or after tDCS. Most subjects reported no difference between active and sham conditions, although 65% of times they guessed the condition correctly. The mean valence ratings for the overall set of pictures was 4.86 (SD = 0.36), being 2.11 (0.57) for negative pictures, 5.40 (0.48) for neutral pictures and 7.32 (0.62) for positive pictures. The mean reaction time was 2.38 seconds (0.38).

The VASs revealed no significant main effects or interactions of type of tDCS (sham or active tDCS) or day of testing (day 1/day 2) in any of the scales except for decreased nervousness following tDCS sessions in either sham or active conditions. In other words, subjects were less nervous once they had completed any tDCS session as compared to before starting the stimulation session, but this was not related to the type of stimulation received (sham or active). No significant effects of stimulation or interactions were observed for the PANAS and STAI-S measures (Table 2).

**Table 2:** Mean scores (and SD or p values) on mood variables (STAI, PANAS and VAS questionnaires).

	Pre Sham	Post Sham	Pre Active	Post Active	F(p) tDCS	F(p)moment	F(p) tDCS*moment
**STAI-S**	13.88 (9.16)	14.12 (9.29)	12.60 (9.60)	11.33 (9.37)	2.84 (0.11)	0.73(0.41)	1.33(0.27)
**Positive Affect**	34.56 (5.69)	35.31 (4.21)	35.53 (5.72)	34.07 (5.52)	0.36 (0.56)	0.21(0.65)	3.62(0.08)
**Negative Affect**	13.12 (3.32)	12.75 (4.25)	12.93 (3.69)	12.93 (4.92)	0.15 (0.70)	0.04(0.84)	0.13(0.73)
**Nervousness**	2.13 (1.88)	1.61 (2.00)	2.60 (2.85)	1.29 (1.39)	0.09 (0.76)	6.25(0.03)*	1.27(0.28)
**Sadness**	1.24 (1.39)	1.18 (1.38)	1.14 (1.05)	1.31 (1.70)	0.24 (0.63)	0.03(0.86)	0.55(0.47)
**Happiness**	7.83 (1.18)	7.84 (1.28)	7.68 (0.95)	7.96 (1.41)	0.01 (0.92)	0.28(0.61)	0.34(0.57)
**Hopefulness**	7.01 (1.66)	6.90 (2.23)	6.54 (2.12)	6.58 (2.60)	0.36 (0.56)	0.01(0.94)	0.05(0.84)

Only a significant Pre-Post decrease in nervousness VAS rating was found.

**Figure 3:**
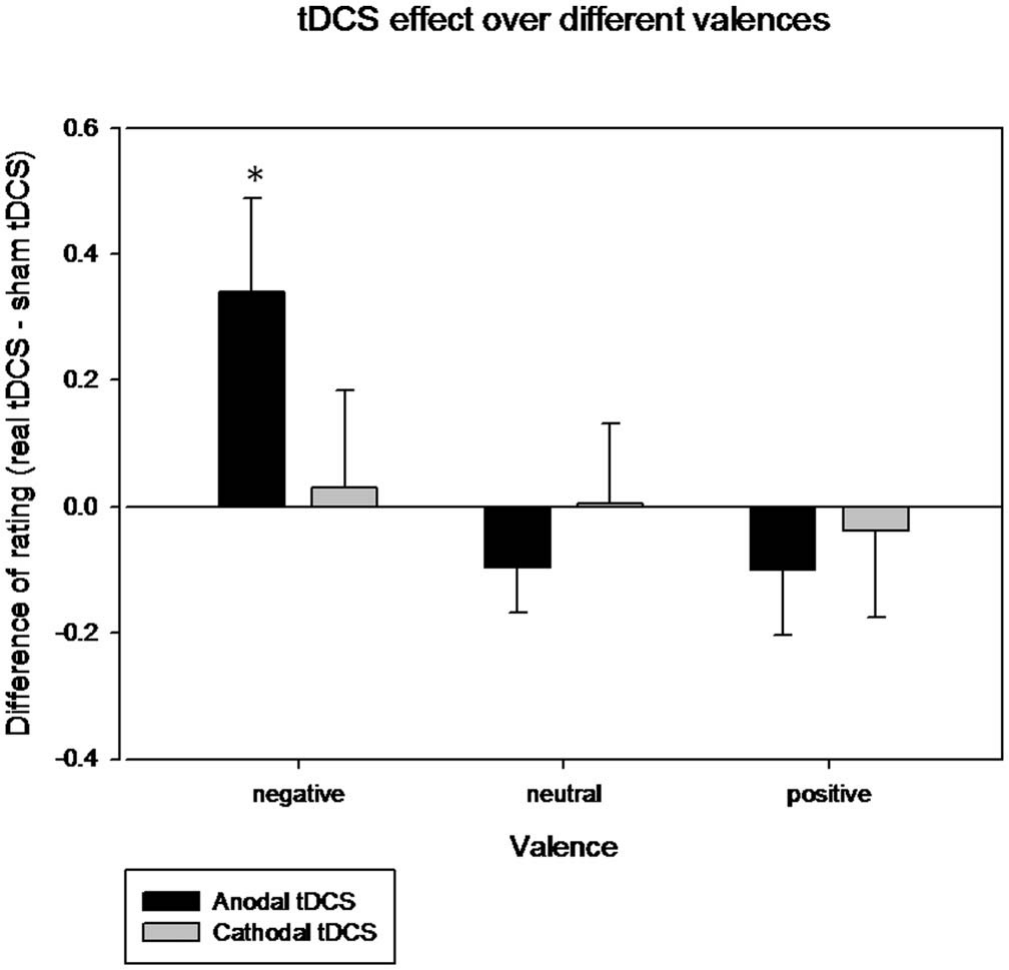
Changes induced by real tDCS in the scores of emotional evaluations compared to sham tDCS. The values correspond to the mean change of rating between sham and real tDCS (and standard errors of mean), both anodal and cathodal, for each valence category. Only ratings for the negative emotional pictures under anodal left DLPFC were significantly different from sham (* p = 0.036, see table 3).

Regarding emotional valence ratings, a significant main effect of valence (F = 428.01; p<0.001) was found, as expected, but neither stimulation condition (F = 0.61; p = 0.45) nor stimulation order (F = 0.38; p = 0.55) were significantly related with emotional rating scores. Regarding the analysis of interactions, a significant effect for the stimulation condition*valence was observed (F = 4.661; p = 0.028), whereas stimulation condition*stimulation order (F = 0.065; p = 0.803), valence*stimulation order (F = 0.741; p = 0.440) and stimulation condition*valence*stimulation order (F = 0.917; p = 0.392) were not significant. Paired t-test comparisons (sham vs. real tDCS) for each level of valence showed that ratings in neutral and positive pictures were unaffected by the condition of stimulation. However, the scores of the evaluation of emotionally negative pictures were higher with real tDCS than with sham stimulation reflecting that subjects perceived these stimuli as being less negative in terms of the degree of their emotional valence (higher values in the scale 1 = negative, 5 = neutral, 9 = positive; see table 3 and figure 3).

Finally, the control experiment with cathodal tDCS over the left-DLPFC only revealed the expected effect of valence (F = 146.012; p<0.001) but no significant effect of stimulation condition (F = 0.004; p = 0.950) or stimulation order (F = 0.763; p = 0.411). No interactions between factors could be observed following cathodal stimulation, including stimulation condition*valence (F = 0.011; p = 0.990). Paired t-test comparisons for the cathodal experiment did not lead to any significant results (see table 3 and figure 3).

**Table 3:** Direct (paired t-test) comparisons for each stimulation condition (active and sham) within each emotional valence (negative, neutral and positive).

		Real tDCS	Sham	T(p)
**Anodal tDCS**	**Negative Pictures**	2.29 (0.67)	1.95 (0.40)	−2.30(0.036)
	**Neutral Pictures**	5.35 (0.46)	5.45 (0.52)	1.37 (0.19)
	**Positive Pictures**	7.28 (0.60)	7.38 (0.66)	0.96 (0.34)
**Cathodal tDCS**	**Negative Pictures**	2.22 (0.65)	2.19 (0.68)	−0.18 (0.85)
	**Neutral Pictures**	5.12 (0.15)	5.12 (0.40)	−0.05 (0.96)
	**Positive Pictures**	4.77 (0.65)	4.75 (0.68)	0.27 (0.78)

Note that only for negative picture ratings there was an effect of real tDCS stimulation (as figure 3 illustrates).

**Figure 4:**
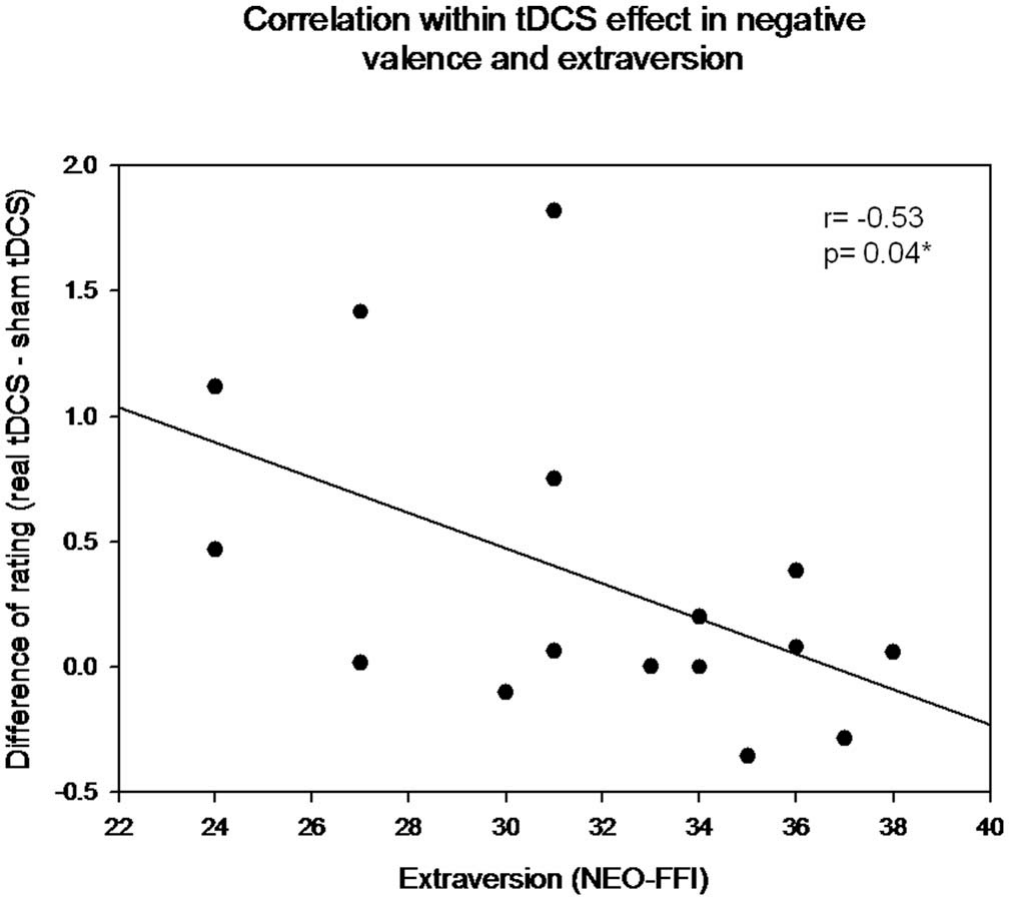
Modulation of personality measures on tDCS effects. The scatterplot depicts a negative correlation between increasing scores of the extraversion dimension of personality (represented in the *x*-coordinate) and the tDCS-induced effects (represented in the *y*-coordinate) evaluated by the mean change of rating in negative emotional pictures between real and sham tDCS. Note that the tDCS modulation is higher the more the subjects manifest themselves as introverts.

As regards personality variables, the mean ratings of neuroticism and extraversion were 21.75 (8.29) and 31.75 (4.44) respectively. Extraversion showed significant correlations with positive picture ratings (r = 0.54, p = 0.03) whereas neuroticism showed a negative correlation with neutral picture ratings (r = -0.61, p = 0.01). There were no significant correlations between neuroticism and extraversion (r = -0.15, p = 0.57). Interestingly, while neuroticism was not related (r = 0.29, p = 0.28) to changes in negative emotional ratings induced by tDCS, a significant effect was observed for extraversion (r = -0.53, p = 0.04). The direction of this correlation indicates that the effect induced by tDCS in decreasing the intensity of negative valence ratings is more evident the more individuals manifest themselves as introvert (figure 4).

## Discussion

The main aim of our study was to investigate whether non-invasive electrical stimulation of the DLPFC could result in a detectable change in emotion regulation processes in healthy young women. Two principal results emerged: (1) Compared to sham and cathodal stimulation, 20 minutes of 1mA anodal-tDCS over the left-DLPFC resulted in increased ratings during negative emotional picture processing, reflecting that such items were perceived less negatively. These results were not explained by more general mood or anxiety changes; (2) This effect was stronger in individuals with higher subclinical scores on the introversion personality dimension.

In general, our results are in agreement with prior studies linking activity of the left frontal lobes with emotional positive mood states [55], [56], including the repeated findings of an antidepressant effect after high frequency rTMS over the left prefrontal cortex [57]. Particularly, our results corroborate those of a previous tDCS study by Boggio et al. [43], which found that applying anodal tDCS over left-DPLFC in healthy subjects resulted in a less unpleasant perception of images demonstrating human pain. Thus, while our results confirm that tDCS is able to modulate the perception of negative emotional stimuli, they also expand on previous observations indicating that this effect seems to be less noticeable for neutral or positive stimuli, and that it is influenced by individual personality characteristics.

The underlying functional brain mechanisms that account for the behavioral effects observed may be multiple and difficult to elucidate. However, since the neural bases and psychological processes underlying emotion regulation have been extensively investigated [9], [10], [11], and since a body of knowledge is now available as regards the behavioral and physiological effects of anodal tDCS stimulation over the DLPFC ([38]–[42], and see introduction section), it is possible to draw some interpretations.

The DLPFC (the PFC region presumably targeted by anodal tDCS in the present study) is a pivotal area of cognitive control mechanism [58], and is associated with cognitive processing when attention must be focused on the external environment [58]. Particularly, it is one of the key prefrontal areas implicated in cognitive control of emotion mechanisms [59] such as reappraisal (including the reinterpretation of the negative situation and distancing one's self from aversive stimuli) and distraction [15], [16], [60], [61]. Hence, as anodal tDCS is thought to facilitate activity in the underlying cortex [30], [34], [38] a plausible interpretation of our behavioral findings may be that tDCS facilitated enabling processes allocated to goal-directed or attentional networks perhaps competing with emotion processing resources, reducing the impact of a vivid emotional experience and resulting in lower scores for emotional ratings. This assumption is indirectly supported by our previous fMRI study which showed that applying 20 min of tDCS over DLPFC increases the temporal synchrony of the fronto-parietal attention system [37]. The interpretation also fits in with the more general notion of a bidirectional competition between goal-directed and emotional processing systems (i.e while emotional stimulus processing often boosts attentional systems, emotional distracters may disrupt goal-directed processing and viceversa; [61]). Specifically, studies investigating the control of affective and cognitive (i.e. non-affective) conflict indicate that the DLPFC (and the lateral parietal in some studies), the posterior medial frontal cortex and the dorsal anterior cingulate cortex are active during conflict between affective and cognitive tasks [61], [62]. However, the DLPFC may be involved more during cognitive conflict monitoring, and the medial frontal cortex during emotional conflict [62]. This latter finding adds further evidence that increasing the cortical activity in the DLPFC area in our study may have biased brain processing resources towards more ‘cognitive’ aspects of the image presented in detriment of emotional processing, thus resulting in more ‘neutral’ ratings of negative stimuli.

The second main finding of our study is that normal variations of the extraversion-introversion personality dimension condition the effects of tDCS on emotional ratings. In spite of the consistent data showing that patients with clinical diagnoses of anxiety-related disorders exhibit deficits in emotional regulation [8], less evidence is available of the impact of specific personality measures on this process [63], and even less when considering subclinical variability in healthy individuals [64]. However, in agreement with our report, Hofman and Schutter [65] recently provided the first evidence that TMS can reveal functional asymmetries between the left and the right hemisphere related to the degree of aggressive personality style and to higher attentional bias scores toward angry facial expressions.

Using tDCS instead of TMS, in the present study we observed that the more introvert a given subject the more permeable she was to the effects of electrical stimulation on emotional regulation. Individual differences in cognition, behavior and emotions as a function of the introversion-extraversion continuum presumably correspond to distinct characteristics of brain functionality. As classically proposed by Eysenck [66], extraverts may have a relatively lower level of cortical arousal associated with diminished activity in retuculothalamic-cortical pathways [67]. Conversely, according to Eysenck's and Gray's theories and subsequent functional neuroimaging findings [25], introverts may have higher activity than extraverts, especially in the frontal lobes.

These observations in the field of personality studies recall the principle of state-dependency, a concept with a long history in psychology and increasingly invoked in interpretations of recent brain stimulation investigations. Here, state-dependency refers to the observation that it is necessary to consider the baseline or ongoing activation state of the targeted neurons as well as the stimulation parameters to predict the response of a system to an external stimulus [68]. As an example, Bestmann and coworkers (2008) [69] used TMS concurrently with event-fMRI to show that state-dependency influenced the interplay between the dorsal premotor cortex and contralateral homologous region and M1. Those authors demonstrated that stimulation of the dorsal premotor cortex while subjects were performing an ipsilateral grip task increased brain activity in the contralateral homologous area as well as in contralateral M1, whereas stimulation in the no-grip rest condition had the opposite effect.

The phenomenon of stochastic resonance may be important in explaining the state-dependency findings in brain stimulation studies, and may provide a mechanistic explanation for our observations. Stochastic resonance refers to the delicate balance between low levels of noise added to a system which has a measurement threshold and the behavioral outcome obtained [70]. In a system of this kind, information transfer is enhanced by the injection of low levels of noise, which lower its response threshold. Hence, if the system's signal strength is subthreshold, adding noise (for example by TMS or tDCS) might make neurons more sensitive to a given range of weak inputs and push them beyond the threshold, leading to behavioral changes. In contrast, if the baseline neural signal of the system is already suprathreshold, then the scope for facilitation is naturally limited and the addition of low levels of noise may have no behavioral consequence. This has been demonstrated in adaptation paradigms of the visual system, where online TMS facilitated motion detection after V5/MT had been suppressed by offline 1 Hz rTMS [71], presumably because the amount of noise being added was lower after adaptation than at baseline. In addition, a recent study [72] has shown how at low intensity online TMS facilitated the detection of weak motion signals, but with higher TMS intensities and stronger motion signals resulted in impairment in detection. The authors concluded that online TMS can induce stochastic resonance in the human brain.

In the framework of stochastic resonance, an explanation for the results presented here is that introverts with higher levels of activity at baseline [66], [67] in combination with a relatively weak neuromodulator technique such as tDCS at a low level of stimulation (1mA current intensity), could have reached the threshold more easily than extraverts, resulting in more marked behavioral changes as subjects had higher scores in this personality trait. In contrast, extraverts would need higher stimulation to the DLPFC for down-regulation of negative emotional processing to ensue. This interpretation is in agreement with Eysenck's classic biologically based theory and with subsequent findings from psychophysiological [73] and neuroimaging studies [23], [25], which have shown that introverts have enhanced sensitivity under low or moderate levels of stimulation, whereas extraverts are expected to manifest higher reusability only under high stimulation conditions [67].

Unexpectedly, neuroticism ratings did not modulate the tDCS effects observed in our study. To our knowledge, the differences in baseline activity levels previously reported for the introversion-extraversion dimension have not found experimental support in the case of the neuroticism dimension. Therefore, the differential effects of state-dependency discussed above may not apply when considering this personality dimension. On the other hand, earlier reports showed that anxious individuals tend to up-regulate their negative emotion states [44] and have difficulty in reallocating attentional resources away from these negative ruminations. Here, before tDCS we found a significant negative correlation between neutral picture ratings and neuroticism that was not maintained after tDCS, where ratings were more adjusted to the standardized neutral ratings. However, no significant association was observed before or after tDCS between negative emotional pictures and neuroticism. Hence, it is possible that the attention resources in subjects with higher neuroticism might be easily deviated from ruminations on negative emotions when they are rating neutral pictures, but emotional regulation would be more difficult during negative picture ratings due to the strong bias in these subjects to upregulate emotions of this kind.

Our study has some limitations that should be overcome in further research. First, despite being a cross-over, sham-controlled investigation, the sample size is relatively small and the findings must be replicated in larger cohorts. Second, as it administered using commonly available electrodes, tDCS is a technique with low spatial resolution. When positioning our anodal electrode over F3, the areas under direct stimulation are grossly the superior and parts of the middle frontal gyri (lateral parts of BA 9, 10), which have been implicated in emotion regulation in neuroimaging studies [60], [61]. However, the effects observed might also respond to a similar mechanism related to the direct modulation of excitability extending to more inferior-lateral areas of the prefrontal cortex (i.e. lower dorsal middle and ventrolateral PFC) where its activity and connectivity with subcortical structures such as the nucleus accumbens and the amygdala positively correlates with behavioural reappraisal [10]. Finally, we can not rule out the possibility that changing the excitability of the DLPFC activity may have trans-synaptically modulated the response of other cortical areas not directly located under the area of the electrode but included in the emotional regulation circuit such as the medial frontal cortex, or even inferior parietal areas [15]. Overall, then, we acknowledge that due to the complexity of the emotional regulation networks and the use of a low spatial resolution technique without concomitant electrophysiological or functional neuroimaging information, the interpretation of our results at the level of the putatively involved neurophysiological areas and connections remains tentative.

In summary, our findings represent the first evidence that increasing the cortical excitability by anodal, but not by sham or cathodal, tDCS of the left-DLPFC results in down-regulation of the ratings of negative emotional stimuli compared with its effect on neutral or positive pictures. Interestingly, this effect is modulated by individual subclinical personality ratings in the introversion-extraversion dimension. Our results confirm and expand the role of the left-DLPFC as a core hub of the emotional regulatory circuit, and open up new possibilities for the use of non-invasive brain stimulation as an add-on treatment that takes account of individual personality differences in patients in conditions where control mechanisms of affectively charged stimuli are compromised, such as chronic pain, anxiety, specific phobias or posttraumatic stress disorder.
